# Degradability of Polymers for Implantable Biomedical Devices

**DOI:** 10.3390/ijms10094033

**Published:** 2009-09-11

**Authors:** SuPing Lyu, Darrel Untereker

**Affiliations:** Medtronic Corporate Science and Technology/710 Medtronic Parkway, Minneapolis, MN 55432, USA; E-Mail:darrel.untereker@medtronic.com (D.U.)

**Keywords:** biostable polymers for implantable medical devices, biodegradable polymers, molecular mechanisms of hydrolysis, biological oxidation, physical biodegradation

## Abstract

Many key components of implantable medical devices are made from polymeric materials. The functions of these materials include structural support, electrical insulation, protection of other materials from the environment of the body, and biocompatibility, as well as other things such as delivery of a therapeutic drug. In such roles, the stability and integrity of the polymer, over what can be a very long period of time, is very important. For most of these functions, stability over time is desired, but in other cases, the opposite–the degradation and disappearance of the polymer over time is required. In either case, it is important to understand both the chemistry that can lead to the degradation of polymers as well as the kinetics that controls these reactions. Hydrolysis and oxidation are the two classes of reactions that lead to the breaking down of polymers. Both are discussed in detail in the context of the environmental factors that impact the utility of various polymers for medical device applications. Understanding the chemistry and kinetics allows prediction of stability as well as explanations for observations such as porosity and the unexpected behavior of polymeric composite materials in some situations. In the last part, physical degradation such interfacial delamination in composites is discussed.

## 1. Introduction

The unintended degradation of polymers often limits the performance of these materials in medical device applications. However, there are cases where degradation is a necessity. Different products may require different stability/degradation properties. For example, polymers used to make chronic implantable biomedical devices have to be stable in biological environments so that the devices perform their functions for a period that can be many years. On the other hand, polymers for tissue engineered implants may need to degrade within a time frame that is comparable to the tissue healing processes (weeks to years). Polymers for drug delivery applications may need to degrade within days to years. Recently, a lot of efforts have been focused on making polymers from natural resources and recycled materials. For these materials, degradation not only affects how they can be used but also how they can be made and processed.

Making biomedical devices involves the use of broad variety of polymers. Ultrahigh molecular weight polyethylene is used to make acetabular cups of total hip replacement prostheses; polyurethane is used in cardiac pacing and defibrillation devices; acrylic polymers are used to make intraocular lenses; silicone is used for breast filling and catheters; polylactide is used for degradable orthopedic rods, screws, and plates as well as drug delivery products; poly(lactide-co-glycolide) (PLGA) is used for degradable sutures; processed pig heart valves are used to replace diseased human heart valves, and so on. In addition to these implantable devices and products, there are a number of engineering polymers used to make surgical tools and supplies.

The duration that a polymer can maintain its designed functions under use conditions is the most important measure of its properties. This duration is called “functional time.” If a polymer is used in a biodegradable device, the time for the materials to completely degrade and lose mass is also important. This time is referred to as “disappearance time.” From function time through disappearance time, a material loses its functionality but still releases degradation products. Depending the chemical and physical properties and release rates, the degradation products can cause biocompatibility concerns. In order for a polymer to be successfully used, the functional time, disappearance time, and degradation products and release rates have to be well characterized and controlled. To do this, one has to understand its degradation mechanisms.

The major degradation mechanisms for the materials used in all these applications are similar, but differ in kinetics due to different processing conditions and use environments. There are four major degradation mechanisms for polymers used in biomedical devices: hydrolysis (reaction with water in tissues), oxidation (due to oxidants produced by tissues), enzymatic degradation, and physical degradation (*e.g.,* water swelling and mechanical loading and wearing). Hydrolysis has been studied extensively, especially for biodegradable polymers. Polymers that degrade via this mechanism include polyesters, polyorthesters, polyanhydrides, polycarbonates, and polyamides. There are several nice reviews of these polymers [1–5]. Oxidative degradation of biomedical polymers was not been fully appreciated until it was observed that a polyurethane material in an implantable electrical insulation lead failed its functions due to this mechanism [6]. One oxidation mechanism is a result of the biological defense action in which inflammatory cells generate oxidative agents that diffuse into polymeric implants and degrade them. Enzymatic degradation is also due to a defensive action against implanted foreign materials. Collagens, polysaccharides (hyaluronic acids), some polyesters (*e.g.,* polyhydroxyalkanoate, PHA), synthetic polycarbonates and proteins are mainly degraded due to this type of reactions [7]. The enzymes come from the biological systems of patients. Hence, due to differences among individuals, the resulting degradation can be different from person to person. For the same person, degradation can be different from tissue to tissue and vary over time. Physical degradation is mostly due to mechanical friction associated with motion under pressure, for example the wearing of acetabular cups of total hip replacements. Water swelling is another mechanism. This can be a problem if swollen polymers have significant changes in glass transition temperature, geometry, and mechanical properties such that the normal functions of the materials are affected.

Chemistry has been the main focus in the reported studies of polymer biodegradation, but chemistry may contribute only part of the processes. Physics plays significant roles as well. For example, polyesters hydrolyze slowest in pH 4 solutions. However, the degradation rates of solid polylactide samples were almost the same in pH 0, 4, and 7. This is because the ions (H+ and OH-) from the test solutions have very low solubility in polymers so they cannot effectively affect the degradation. There are many other examples that cannot be simply explained based solely on chemistry. However, they can be well understood if transport, solubility, and other physical processes are considered together. But combining chemical and physical approaches to study degradation is challenging. This often involves solving diffusion-reaction equations. In addition, there are some practical issues in the studies of degradation of polymers; samples for many studies were in forms such as micro particles, gels, or tubing that have various geometry, packing properties (porosity versus solid), processing history (orientation, amorphous, and crystalline), and measurement targets. Because transport and solubility are sensitive to those issues, it is difficult to correlate the results to each other.

This review will follow a line of combined chemistry and physics. It will focus on ideal samples (*e.g.,* thin films) instead of “real” products such as drug delivery particles. Hydrolysis, oxidation, and physical degradation will be discussed. For each mechanism, the discussions will be from molecular mechanism to macroscopic processes. In the last part, polymers used in medical devices are discussed.

## 2. Hydrolytic Degradation

Hydrolysis is a major degradation mechanism in which vulnerable bonds in a polymer chain react with water molecules, break up, and result in smaller chains. Chemical reactivity of polymer bonds, diffusion rates of reactants and products including water, polymer bonds, ions in testing media, and small polymer segments, and polymer-water thermodynamic interactions all are involved. In order to understand each fundamental step of polymer hydrolysis, studies should be designed such that these factors are decoupled. In this review, hydrolytic degradation will be discussed at three levels to decouple those factors. The first part is at the molecular level. Polymer solutions are used so the diffusion of reactants and the polymer-water interactions are not limiting factors. Therefore, hydrolysis is controlled by chemical reactivity only. A second part also is at the molecular level but the molecular mobility and water-polymer interactions are added to the degradation processes by using bulk samples. A third one is at macroscopic level. The focus is on the diffusion-reaction competition at the macroscopic scale. Surface erosion is at this length scale and is discussed. In all these discussions, a polylactide sample (PLA) composed of 70% l-lactic acid and 30% d,l-lactic acid is used as the example. Other polymers such as poly(anhydride), polyorthoesters, and poly(amino acid) may have different degradation kinetics, but the same principles should apply.

### 2.1. Hydrolysis in Solutions and Gels–Chemistry of Hydrolysis

#### 2.1.1. Chemistry of Hydrolytic Decomposition of Polymers

**Scheme 1.**
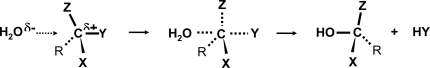
Schematic of hydrolysis. This reaction is a 2^nd^ order nucleophilic substitution reaction. The reaction group breaks and two new species form.

In hydrolytic degradation, polymer bonds react with water molecules, break up, and produce new chain ends. The original chains break up into smaller segments, resulting in polymer degradation. Most chemical groups that react with water contain O, N, S, P, and other non-carbon atoms. These atoms cause the adjacent carbon atoms to be positively charged. Electron-withdrawing oxygen atoms of water molecules attack these positively charged carbon atoms through a 2nd order nucleophilic substitution reaction resulting in the formation of two new chemical spices. A general reaction mechanism is illustrated in Scheme 1.

The reactant molecules, X, Y, Z, and R, can be C, O, N, and other atoms. The charge value of the reacting C atoms is a primary factor affecting the hydrolysis reactivity. Charge values of a few common groups have been calculated using Accelrys Material Studio® and are listed in Table 1. The chemical groups such as esters, anhydrides, orthoesters, etc. are usually more susceptible to hydrolysis than C-C, C-O-C moieties, etc. The charge values of these two types of chemical groups seem to be distinguished by a value of 0.3 electron charges. The chemical groups having charge higher than 0.3 electron charges seem to be hydrolytically more active than those with charge lower than 0.3. However, hydrolytic activities of chemical groups also depend on many other factors such as conjugate structures that stabilize chemical groups. For example, urethane, carbonate, and aromatic ester groups have large conjugate structures; so they are generally more stable than aliphatic esters against hydrolysis, even when their charge values are high. The side groups also reduce hydrolytic activity of carbons via steric effects. For example, the silicon atoms in polydimethyldiloxane (PDMS) have 0.62 electric charges, but the two methyl groups closely protect the silicon atoms and PDMS is rather stable towards hydrolysis. For the same reason, poly(lactic acid) (PLA) is less reactive than poly(glycolic acid) (PGA). On the other hand, polyorthoester, even with a relatively low charge at its central carbon atoms, reacts with water rather fast. This is due to the high bond tension caused by the three RO- groups around the carbon atoms. Besides these, the dielectric constant, degradable bond concentration, and water solubility also affect the hydrolytic activities of polymers. Overall, all chemical groups, except pure C-C bond, can undergo hydrolysis reaction. The only difference is that they react at different rates.

#### 2.1.2. Kinetics of Polymer Hydrolysis

The hydrolysis of polymer bonds follows 2^nd^ order reaction kinetics, *i.e.,* the rate of the reaction is proportional to the concentration of water and hydrolytic polymer bonds. The hydrolysis products are chain ends, but the chain end concentrations (E) usually are very low and their direct measurement is difficult. Chain end concentration is reciprocal to the number average molecular weight of polymers (Mn). Measurement of molecular weights is routine for polymer materials, therefore hydrolysis kinetics are often characterized by molecular weight as a function of time. Using polyesters as examples, we have the following relationships. The chain end concentration is related to molecular weight by Equation (1):

(1) $$[E]=\frac{\sum }{V}=\frac{\rho }{Mn}=\frac{\rho }{N\cdot M_{0}}$$

where Σ, V, W, and ρ are the total number of chains, volume, weight, and density of the samples. N is the degree of polymerization. M_0_ is the monomer molecular weight. Mn is number average molecular weight. Since hydrolysis of polymer bonds is a second order reaction, we have:

(2) $$\frac{dE}{dt}=k_{2}C_{B}C_{S}$$

where $C_{B}=\rho /M_{0}(1-1/N)$ is the total bond concentration and *C_S_* is the water concentration. Combining Equations 1 and 2 and substituting N>>1, we have:

(3) $$\frac{1}{Mn}=\frac{1}{Mn_{0}}+\frac{1}{M_{0}}k_{2}\cdot C_{S}\cdot t$$

This says that if degradation is a 2^nd^ order reaction, 1/Mn should have a linear relationship with time. In certain cases, the chain ends are acids and catalyze the hydrolysis. This is the so-called autocatalysis or auto-accelerated degradation. In this case, the reaction rate is proportional to the chain end concentration as well as water and hydrolytic bond concentrations, and we would have:

(4) $$\log\;(Mn)=\log\;(Mn_{0})-k_{3}\cdot C_{B}\cdot C_{S}\cdot t$$

By fitting experimental data to Equations 3 and 4, one can determine whether the reaction is a 2^nd^ order hydrolysis or autocatalysis [8].

#### 2.1.3. Effects of Polymer Concentration and Chain Mobility on Hydrolysis Kinetics

Solid polymers, concentrated solutions or gels, and dilute polymer solutions have different concentrations of hydrolytic bonds. In solid polymers, the bond concentration is uniform throughout the entire sample. In concentrated solutions in which polymer chains overlap with each other, the bond concentration is also uniform although it can be very low. In dilute polymer solutions where individual polymer chains form coils dispersed in the solvent, bond contraction inside coils is equal to *C_in–coil_* = *N*/(*N*^3α^*a*^3^) = *N*^1–3α^*a*^−3^ but zero outside (α is constant from 0.5 to 0.6 and *a* is a function of monomer size). Therefore, dilute systems are no longer uniform. How does bond concentration affect degradation rates in these different systems? Interestingly, the effects are not significant.

**Figure 2.**
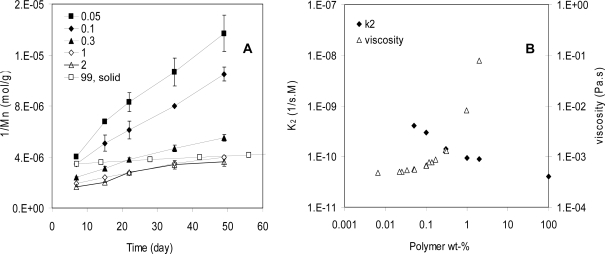
A, 1/Mn versus time for PLA in tetrahydrofuran (THF) solutions and solid samples. Dilute solutions include those with 0.05 and 0.1 wt% of polymer where individual polymer chains dispersed in solvents. Concentrated solutions include those with 0.3 to 2 wt% polymer where polymer chains entangled with each other. The solid sample contains 99 wt% polymer. All the samples contained 1 wt% of water (made by adding 1 wt% of water to the PLA solutions in THF). B, hydrolysis rate constant k_2_ and viscosity of solutions versus polymer concentration. The viscosity is reciprocal to the molecular mobility.

Figure 2A shows the degradation results of a poly(l-lactide-co-d,l-lactide70/30) (PLA) in tetrahydrofuran (THF) and of solid polymer samples. The concentration of PLA varied from 0.05 wt% in the dilute solution to 99 wt% in the solid, spanning a range of about 2,000-fold. The degradation rate (k_2_) of PLA in dilute solutions was higher by a factor of 10 compared to that in the solid. On the other hand, the changes in polymer chain mobility were dramatic. The polymer chain mobility was measured by the viscosity of the samples because mobility is reciprocal to viscosity of the systems. From the dilute solutions to concentrated solutions (2 wt%), the mobility of the PLA was reduced by a factor 100 (Figure 2B). The reduction in mobility from the concentrated solutions to the solid should be much more than 100 fold because the viscosity of the solid is too high to measure. These results indicated that although the PLA chain mobility was reduced by much more than 100 fold, its degradation rate decreased by just a factor of 10. Therefore, chain mobility does not affect the hydrolysis rates of polymers very much. In other words, hydrolysis is controlled by the chemical reaction but not molecular mobility of these systems.

#### 2.1.4. Effects of Water Concentration on Kinetics of Hydrolysis in Solutions

**Figure 3.**
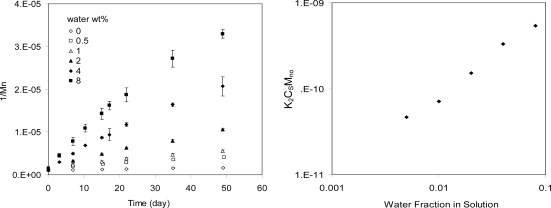
Effects of water concentration on hydrolysis of PLA in THF solutions. A, 1/Mn versus time. B, slope of the hydrolysis curve in A (k_2_C_s_/M_n0_) versus water concentration. The linear relationship suggests the proportional dependence of hydrolysis of PLA on water concentration.

Hydrolysis rate of polymers should be proportional to water concentration. There have been very few experimental studies to directly confirm this, especially for water insoluble polymers such as polylactide and other polyesters. One direct experimental measurements was also made with poly-(l-lactide-co-d,l-lactide 70/30; Mn = 300 kg/mol). This polymer is not soluble in water. It was dissolved in tetrahydrofuran (THF) at low concentration (0.3 wt%). Various amounts of water were added to the solutions. The degradation results are shown in Figure 3. 1/Mn is nearly proportional to time, which indicates the hydrolysis follows 2^nd^ order kinetics. Based on Equation 3, the slope of the curve is k_2_C_s_/M_n0_. This slope was plotted as a function of water concentration C_s_. Hence, the proportional relationship between the hydrolysis rate and water concentration was demonstrated (Figure 3B).

#### 2.1.5. Effects of pH on Hydrolysis Kinetics in Polymer Solutions

**Figure 4.**
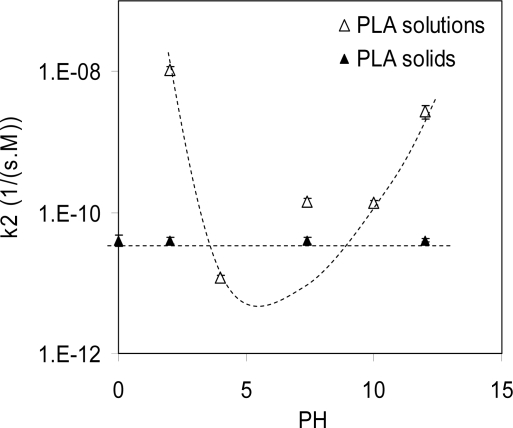
Effects of pH on the hydrolysis of PLA in solutions. The slowest degradation occurred in the solution of pH 4. Notice that the degradation rate of solid PLA solid (solid triangles) is almost independent of the pH of testing media.

The effect of pH on the hydrolysis reaction of polymers has been studied in a few reports. Figure 4 show the hydrolysis of PLA in THF solutions with nominal pH varying from 0 to 14. As shown, the slowest reaction rate was at pH 4. This is because the PKa of lactic acid is 3.84 [9]. In a solution with pH > 4, the lactic acid is mainly in the dissociated form, which accelerates the hydrolysis. In solutions with pH < 4, lactic acids at the chain ends prefer the associated acid form, which also accelerates the hydrolysis reaction via auto-acceleration. Therefore, the degradation rate of polymer solutions is the slowest at pH 4. However, similar effects of pH on degradation have not been observed in solid polymer samples. This is because the ions such as H^+^ or OH^−^ have very low solubilities in solid polymers, so they cannot diffuse readily into the solid samples to affect the bulk hydrolysis. As shown in Figure 4, the hydrolysis rates of solid samples (solid triangles) are not sensitive to the pH of the media.

#### 2.1.6. Effects of Temperature on Degradation Rates

**Figure 5.**
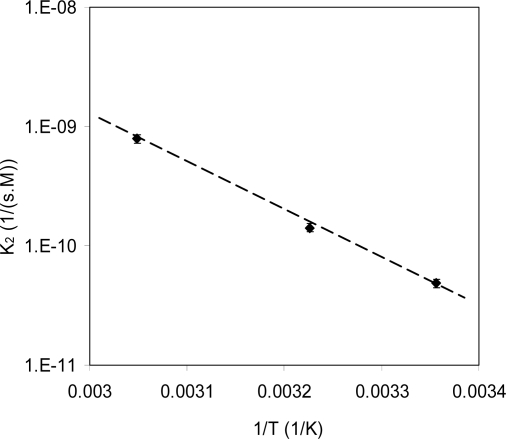
Hydrolysis rate constant as a function of 1/T (T is the absolute temperature). The linear relationship indicates the degradation process is activation controlled. The slope indicates an activation energy of 76 KJ/mol.

Hydrolysis rates of polylactide in THF solutions fit the Arrhenius equation. Figure 5 shows the degradation rate constant (k_2_) of PLA in THF solution (0.3 wt%) as a function of the reciprocal of testing temperature. The linear curve indicates the degradation of PLA in solution is controlled by activation energy. The activation energy in this case was 76 KJ/mol (in a temperature range from 25 to 50 °C).

#### 2.1.7. Polydispersity of Degrading Polymer Chains in Solutions and Solids

**Figure 6.**
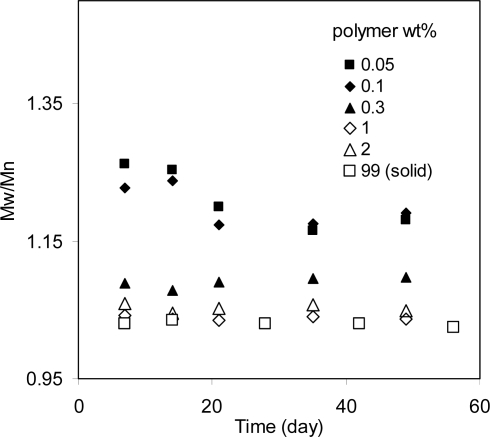
PDI of degrading PLA in THF solutions and solids. The PDI either did not change or decrease, indicating that all the bonds have equal reactivity in this test.

There is a debate about how the hydrolytic activity of a polymer bond is related to its position in a polymer chain. Some authors have suggested that the bonds near chain ends have higher activity [10] while others argue that all the bonds have equal activity and they degrade randomly. One experiment that may determine whether all polymer bonds have equal reactivity is to measure the polydispersity index (PDI) of the molecular weight distribution. A larger polymer chain coil has more bonds than a smaller one. If each bond has equal reactivity, the larger chain would have a higher likelihood of reacting with water and more quickly degrading into smaller chains. For analogous reasons, smaller chains should degrade slower. As a result, the polymer chains tend to become similar in size, or a PDI approaching 1. If the bonds near the chain ends degrade faster, smaller chains would degrade relatively faster than larger ones and the PDI of whole samples would increase as a function of time. Figure 6 shows the PDI of PLA degraded in solutions of different concentrations (with 1 wt% of water). The PDI of dilute solutions decreased. For concentrated solutions or solid polymer, the PDI did not change significantly with time. In these samples, the Mn decreased by almost 50%. If the bonds near chain ends are responsible for these molecular weight changes, the PDI should have a significant increase instead of no change. Therefore, these results suggest that the all the bonds have equal reactivity within these testing periods.

#### 2.1.8. Summary of Polymer Hydrolysis in Solutions

The hydrolysis of polymers in their solutions is controlled by chemistry instead of diffusion. The reaction kinetics is similar to that of small molecules. Hydrolysis is a 2^nd^ order nucleophilic substitution reaction; the reactivity is mainly correlated with the electric charge of the reacting carbon atoms although steric effects and conjugated structures also have effects. The reaction rate is proportional to polymer bond and water concentrations. For polyesters, the hydrolysis rate has a minimum at pH4. It seems all the bonds of a high molecular weight polymer chain have equal reactivity.

### 2.2. Hydrolysis in Solid Polymers–Bulk Degradation

Polymers are often used in solid forms. Solid polymers differ from polymer solutions in several important ways in terms of degradation. Most obviously, molecular mobility in polymer solids, either glassy or rubbery, is lower than that in polymer solutions, and molecular mobility may affect hydrolytic degradation. In cases where mobility controls the reaction rate, the kinetics would depend on the diffusion rate of the reactants instead of the hydrolysis rate constant (*k_2_*) (in diffusion controlled cases, the kinetic constant is proportional to the diffusion coefficient and reaction volume size as described by the Smoluchowski equation) [8]. Secondly, in polymer solids, water concentration is a constant and determined by the water solubility in the polymers. Typically, polyesters (*e.g.,* PLA) can absorb about 1 wt% of water. As molecular weight deceases due to hydrolysis, more hydrophilic chain ends are made and samples may absorb increasing amounts of water. There are two critical values of polymer molecular weight that relate to these properties. One is the molecular weight above which polymers have acceptable mechanical performance. This is referred as functional molecular weight (*M_f_*). This functional molecular weight depends on both polymer properties and application needs. The other is the soluble molecular weight (*M_s_*). Polymer chains become water soluble when their molecular weight is lower than *M_s_*. The soluble molecular weights are determined by thermodynamics of the polymer and water system. In the following section, these questions will be reviewed.

#### 2.2.1. Theoretical Analysis of Bulk Degradation in Terms of Reactions and Diffusion

As discussed in the previous section, in solution hydrolysis is a 2^nd^ order reaction. However, it appears to be 2^nd^ order only if the diffusion or convection of reactants is faster than the consumption of reactants so the reactants used can be immediately replaced through diffusion such as in gas phase reactions. Diffusion in polymers generally is slow. Therefore, the effects of the mobility of polymer bonds, chain ends, water molecules, and ions (H^+^ or OH^−^) on hydrolysis need to be considered. The following discussion explains observations when hydrolysis in solid polymers (*e.g.,* PLA) is controlled by reaction rate and when it is controlled by a diffusion process.

Diffusion coefficients of water in polymers are not very sensitive to polymer states, such as glassy or rubbery states, or polymer types. The measured diffusion coefficient of water in a crystalline polylactide at 37 °C (<T_g_ = 66 °C) was 10^−8^ cm^2^/s. Interestingly, the water diffusion coefficient in amorphous glassy PLA samples at the same temperature is also around 10^−8^ cm^2^/s (poly(d,l-lactide) and poly(d,l-lactide-co-l-lactide 30–70). The degradation rates of these three PLAs are very different. See Figure 11). This suggests that regardless of whether polymers are crystalline or amorphous, the water diffusion coefficients in polymers are within a relatively narrow range. We can estimate that, in a glassy PLA, it takes water molecules 10^−6^ second to diffuse a distance of 1 nm (about a monomer length), and it takes a few days to diffuse through 1 mm (a typical sample size). The saturated concentration of water in the amorphous PLA is also the same as that in the crystalline one; both are about 1 wt%.

Salts and ions usually have low solubility in polymers. Therefore, ions such as H^+^ and OH^−^ from testing solutions do not have significant effects on the degradation of polymers as they do not migrate into the samples. If there are organic acids in the test media that can diffuse into the polymers, the degradation may be accelerated.

The mobility of polymer chains usually is very low due to their great sizes. In polymer melts (testing temperature much higher than their Tg), diffusion coefficients of a small polymer chain is reciprocal to its molecular weight. If the chain size is larger than the entanglement molecular weight, the diffusion coefficient is reciprocal to the square of the chain size. When the testing temperature is lower than the polymer Tg, the mobility of polymer chains, regardless of their sizes, is essentially zero. Overall, the diffusion coefficients of polymer chains can be 10^−10^ to 10^−18^ cm^2^/s in melt states and lower than 10^−18^ cm^2^/s in crystalline or glassy states [11,12]. Therefore, compared to water molecules, polymer chains have much lower diffusivity and are almost immobile. For this reason, when discussing the effects of diffusion on polymer degradation, we may only consider the diffusion of water molecules.

**Figure 7.**
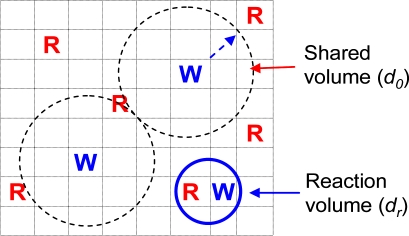
Characteristic lengths in a solid reaction system. *R* represents reactive polymer bonds and *W* represents water molecules. Each water molecule shares a volume of diameter *d_0_*. *R* and *W* have to move into a reaction volume of a diameter of *d_r_* in order them to react.

When we discuss whether the hydrolysis reaction is controlled by chemical reaction or diffusion of reactants, we need to compare a few characteristic lengths and times as shown in Figure 7. One length is the average distance between reactants (initial distance *d_0_*). Each reactant molecule shares a volume with a diameter *d_0_*. Another length is the maximum distance within which the reactants can react. This length is called the reaction radius *d_r_*. Usually *d_r_* is about 0.1 nm. A volume with a diameter *d_r_* is called the reaction volume. Here we use the same polylactide (PLA, 70% l-lactide and 30% d,l-lactide) as an example. Typically, PLA has a density around 1.2 g/cm^3^. The average polymer bond (monomer) distance *d_0_* can be estimated as ~0.6 nm (for example, each polymer bond shares a volume of about 1/[(1.24 g/cm^3^)/(74 g/mol)]/(6 × 10^23^/mol)~0.1 nm^3^, which has a diameter of ~0.6 nm; 74 g/mol is the PLA bond molecular weight). Typically, PLA has about 1% of water when saturated (equivalent to 0.5 M). Each water molecules shares a volume of 3.3 nm^3^ [~1/(0.5 mol/L)/(6 × 10^23^/mol)]. The average water molecular distance is about 2 nm. Because water molecules are well mixed with the polymer bonds, the water-polymer bond distance should be equal to the bond-bond distance (0.6 nm). The chain end distances depend on the molecular weights of the polymers. If a PLA molecular weight is about 200 kg/mol, the average chain end (acid) distance can be estimated similarly and that would be 8 nm. Also, the average chain end distance would be 6 and 4 nm for a PLA with molecular weight 100 kg/mol and 20 kg/mol, respectively.

In order for a PLA bond and a water molecule to react, they first must diffuse from *d_0_*~0.6 nm to *d_r_*~0.1 nm. The time needed for this diffusion is *t_d_*. They would react within an average time *t_r_*. In solid PLA, this diffusion time is *t_d_*~10-7 s (water molecules complete this diffusion because it diffuses much faster than a polymer bond. *t_d_*~(0.6 nm)2/(10-8 cm2/s)~10-7 s). The reaction time is *t_r_*~103–6 s [8]. Comparing the *t_r_* and *t_d_* for this case, one can immediately conclude that the diffusion of reactants in PLA is much faster than the reaction, therefore the hydrolysis in the PLA is controlled by the reaction instead of diffusion. The same conclusion is true for other polyesters.

In crystalline polymers, mobility may be lower than that in amorphous polymers. However, it has been determined that the diffusion rate of water in crystalline polymers is almost identical to that in amorphous polymers of the same chemical compositions. As discussed above, the diffusion of water and polymer chains do not control the hydrolysis rates. This suggests that a crystalline polymer should degrade at the same rate as an amorphous polymer of the same chemistry. Oriented polymer should have similar degradation rates as well. This has been reported in the literature [13].

Salts have very low solubility in polymers with high molecular weights. Therefore, the ions in test media cannot affect the degradation of bulk samples. However, they can affect the degradation of the surface layers [8]. For example, when a PLA solid sample was tested in a solution of pH 12, its surface undergoes much faster degradation than the inside the sample, leading to the sample surface deterioration.

**Figure 8.**
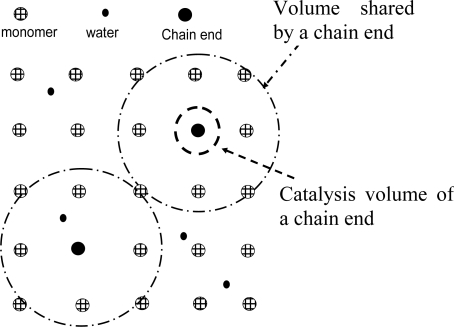
Chain ends E may be able to catalyze the reaction between a polymer bond R and a water molecule W. However, E can catalyze only those R-E pairs that are sufficiently close to a E such that it can diffuse to them. Therefore, only a certain percentage of R-W pairs within the shared volume of E can have an accelerated reaction rate [8].

The acidic chain ends of polyesters tend to accelerate the degradation of the sample (auto-acceleration). A question is what percentage of polymer bonds in a sample can be catalyzed by the chain ends of the sample? In order to answer this question, we need to estimate what distance a chain end can diffuse within a catalyzed reaction time *t_cr_* (Figure 8). Take *t_cr_*~105 s and for chain ends, D~10^−18^ cm^2^/s [8]. One can then calculate that the chain end can explore a volume with a diameter of about 3 nm. For a PLA sample with Mn = 200 kg/mol, the occupied volume of a chain end has a diameter of 8 nm (as discussed above). This suggest that about 5% (=3 nm/8 nm)^3^ of the ester bonds in a PLA sample of 200 kg/mol can be catalyzed by its chain ends. This is not very significant. If Mn = 20 kg/mol, the chain end distance is about 4 nm. Then, almost 40% = (3 nm/4 nm)^3^ of the bonds can be catalyzed. Auto-acceleration in this sample would be significant. These two estimations tell that the effects of auto-acceleration by chain ends are much more significant for either lower initial molecular weight polymers or as the molecular weight decreases during the degradation processes. However, as the molecular weight decreases, the polymer sample can absorb more water which can result in the pH values inside and outside the sample become closer. Auto-acceleration can become insignificant again. Chain ends can also explore greater volumes if their hydrolysis rates are slower. Therefore, auto-acceleration effects can be more significant in polymers that have slower degradation rates (for example polycaprolactone compared to polylactide).

#### 2.2.2. Experimental Observation of Bulk Hydrolytic Degradation of Solid Polymers

**Figure 9.**
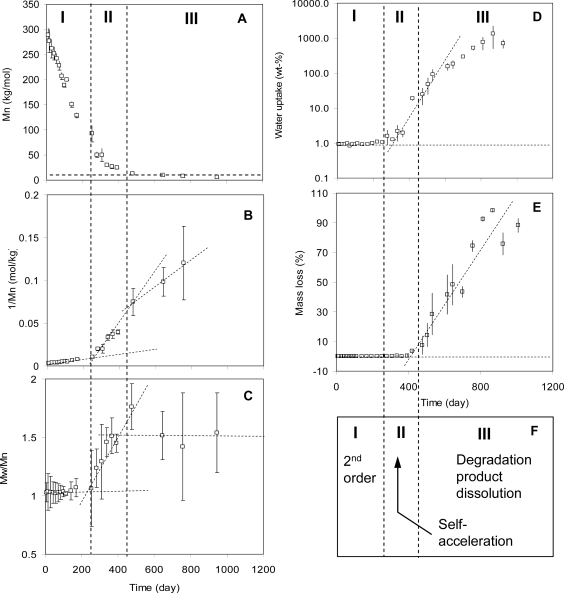
Experimental results of bulk degradation of solid PLA (70% l-lactide-co-30% d,l-lactide) at 37 ^°^C. The degradation has three stages. The first is 2^nd^ order hydrolysis. The second stage is auto-accelerated, and the third is dissolution of degradation products. These stages are shown by the zones separated by the vertical lines in panel (F) [8].

There are three stages in a bulk degradation process [8]. Depending on the type of polymers and their molecular weights, some polymers can go through all the three stages while others may just go through one or two. Figure 9 summarizes all the three stages of a bulk degradation process using the same high molecular weight PLA as an example. When the samples contact water they become saturated quickly (within a few hours to a few days for typical samples with thickness on the order of 10 microns to 1 mm). In the first stage, the polymer bonds react with water molecules with 2^nd^ order kinetics. There is no auto-acceleration. This is characterized by the linear relationship between 1/Mn and time as shown in Figure 9B. Other characteristics of this stage include the PDI does not change significantly with time, indicating most of the bonds react with water at an equal rate (Figure 9C); the samples are saturated with water and the saturated water concentration is almost a constant (about 1 wt% for PLA) (Figure 9D); no mass loss occurs (Figure 9E).

This first stage of the degradation of a solid polymer is similar to degradation in a polymer solution in terms of reaction kinetics (reaction controlled 2^nd^ order kinetics, random degradation, and no auto-acceleration). The main reasons are the molecular weight is high and the chain end concentration is low. Due to this, the auto-acceleration is insignificant and water swelling is low. However, there is one big difference as well. In polymer solutions, water concentration is an experimental variable that can be controlled. In solid polymers, water concentration is determined by the water solubility and cannot be adjusted.

In the second stage, the molecular weight of the polymers decreases and reaches a value such that the concentrations of the chain ends are high enough they catalyze a significant percentage of polymer bonds. Auto-acceleration becomes characteristic of this stage and degradation rate increases (Figure 9B). The polymer bonds that are closer to chain ends (same chains or difference chains) have more chance to be catalyzed than those far from chain ends, leading to heterogeneous degradation and an increase of PDI (Figure 9C).

As the concentration of chain ends further increase, the interactions between the polymer and water changes and the polymer absorbs more water. It is evident in the increased water uptake (Figure 9D). As the molecular weight of the polymer further decreases, it eventually reaches a value such that some of the polymer chains are small enough to become soluble in the testing media. These polymer chains dissolve and mass loss starts. This defines the start of stage 3 (Figure 9E). Because the small molecular weight components continue to dissolve in the testing solutions, the molecular weight of the remaining sample levels off (Figure 9A). The terminal molecular weight value defines the soluble molecular weight of the polymer at which the degraded chains dissolve in the testing media. For PLA, as shown in Figure 9A, the soluble molecular weight is about 5 to 8 kg/mol.

**Figure 10.**
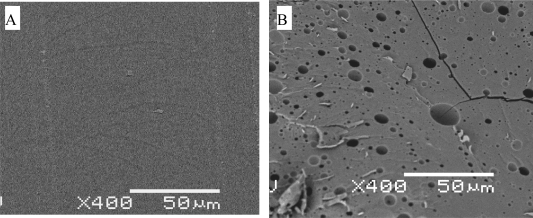
Voids induced by degradation in a solid poly(d,l-lactide 50/50) sample. A, the SEM morphology before degradation. B, the morphology after soaked in pH 7 aqueous solution at 37 °C for 6 months. The initial molecular weight of the sample is about 10–40 kg/mol (courtesy of Lohstreter).

This general picture may vary for several reasons. For example, if the samples are very thick the polymer chains inside samples may not be able to migrate out from inside the sample into the testing media even when they reach the soluble molecular weight. These small chains cause more water absorption and eventually form pockets of solutions inside the sample. The samples then become microscopically porous or form macroscopic water pockets and voids [14]. A morphology with voids induced by degradation in a PLA samples is shown in Figure 10.

On the other hand, when samples are thin, polymer chains with soluble molecular weights can readily diffuse out from samples. The pH inside and outside the samples may become similar. Auto-acceleration effects by these chains will be reduced. It has been reported that small polymer particles degrade slower than larger ones [15]. This probably is due to this reason. If polymer samples are crystalline, the chain ends diffuse slower than that in amorphous ones. Auto-acceleration in small crystals can be minimal.

#### 2.2.3. Effects of Monomer Sequence

**Figure 11.**
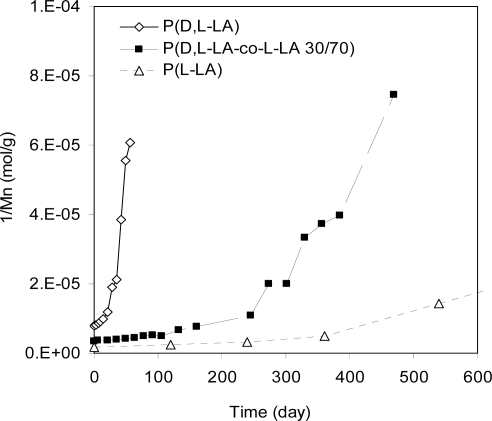
Degradation of crystalline P(l-LA) and amorphous P(d,l-LA-co-l-LA 30/70) and P(d,l-LA 50/50). The reciprocal molecular weights measure the concentration of degradation products. P(l-LA) had slowest increase in reciprocal molecular weight as a function of time, indicating it degraded slowest compared to the other two amorphous materials.

The effect of monomer sequence on degradation rate is more substantial than any other factor. Figure 11 show degradation rates of three different PLAs, a crystalline poly(l-lactide), an amorphous poly(l-lactide-co-d,l-lactide 70/30), and an amorphous poly(d,l-lactide 50/50). As it is shown, the more l-d bonds, the faster the degradation. Similar results were observed in poly(lactide-co-glycolide) copolymers. The conclusion is that the co-polymer bonds have a higher degradation rate than the homopolymer bonds. It is easy to understand why the l-g (G represents glycolide) degrades faster than l-l due to steric effects of the methyl group in l. However it is not clear why d-l degrades faster than l-l The present authors proposed a concept of collective steric effect. It was speculated that two or more consecutive l groups may work together to affect the degradation rate. It is well known that the side groups affect the conformation of the polymer chains such as the 2^nd^ order structure of protein molecules. Also, the consecutive side groups may have collaborative steric effects. The l-l (and d-d) configuration would have different collective effects from the l-d (or d-l), and these differences may be responsible for the different degradation rates.

#### 2.2.4. Effects of Temperature and Accelerated Hydrolysis

**Figure 12.**
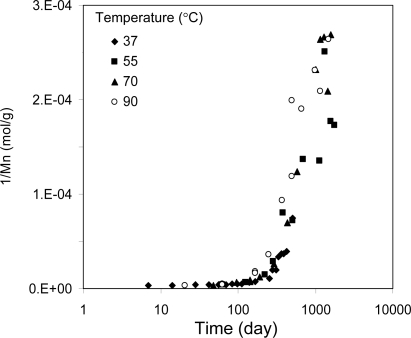
Time-temperature superposition of the degradation of PLA (70% l-lactide-co-30% d,l-lactide) solid samples tested at 37, 55, 70, and 85 °C. The data at higher temperatures overlap with that at 37 °C via the WLF data shifting [8].

Understanding the effect of temperature on the degradation kinetics is important to not only predict the degradation behavior of the material, but also extremely useful to develop an accelerated degradation test method. Some polymer degradation takes more than three years [16]. Testing for such a long period is certainly time-consuming. Deng *et al*. reported an *in vitro* degradation study of PLGA fibers [17]. The degradation was performed over a temperature range of 27.5 to 47.5 ^°^C. Changes in the strain at break were found to obey the Arrhenius equation. Degradation tests at high temperatures (>100 ^°^C) were reported. Arrhenius behavior was observed here as well. These tests were conducted at temperatures that did not cross a glass transition. If degradation is tested from below the Tg to above it, will the degradation still obey the Arrhenius equation? The answer is that the degradation follows the time-temperature superposition or WLF equation if tested from below to above the Tg. Figure 12 show degradation results of PLA tested at 37, 55, 70, and 85 °C [8]. The Tg of this polymer after soaking in water is about 40 ^°^C. The degradation curves at four different temperature overlaps by applying WLF shifting. The degradation of PLGA also obeys the WLF equation [13]. Interestingly, the degradation of blends of PLGA/PTMC also obeys the WLF equation [13].

#### 2.2.5. Bulk Degradation Summary

Degradation of solid polymers is reaction controlled instead of diffusion controlled. Carboxylic polymer chain ends catalyze the reactions between ester polymer bonds and water molecules. However the percentage of the affected polymer bonds is related to the chain end concentration in the samples. Chain end concentrations are lower in samples of higher molecular weights than that in samples of lower molecular weights. Therefore, the auto-acceleration phenomenon is insignificant in high molecular weight polymers.

### 2.3. Surface versus Bulk Erosion

**Figure 13.**
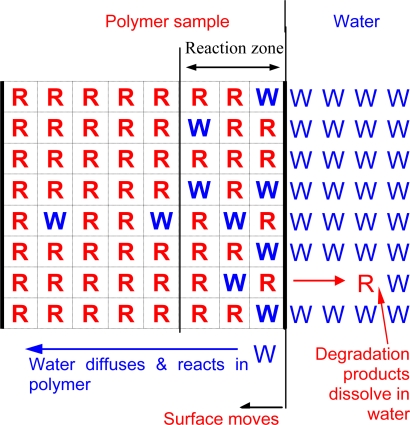
Schematic of surface erosion. There is a reaction zone near the sample surface. Within this zone water concentration follows a gradient and the degradation reaction is controlled by kinetics instead of diffusion at a molecular level. Beyond this zone, the degradation reaction is very slow because the water concentration is low. Diffusion of water affects the degradation reaction at a macroscopic scale but not at a molecular scale.

Surface erosion means that the degradation and mass loss occur within a region near surface and they march into the bulk of samples over time (Figure 13). This is similar to metal corrosion that works its way in from the surface. Compared to bulk erosion, surface erosion could provide more linear changes in mechanical performance, drug release, and volume reduction, and therefore is attractive for many applications.

Surface erosion and bulk erosion are similar at the molecular levels, but different at macroscopic levels. Consider a film sample with a thickness *W’*. It is dry initially and dropped into water at time zero. Two events will occur. One is the water in the test media starts to diffuse into the film immediately. The rate at which the water front moves into the sample depends on the water diffusivity.

The other event is the polymer bonds that contact water molecules start to degrade and the chain molecular weight begins to decrease. Because the polymer bonds at the sample surface contact water molecules first, they degrade the earliest. When the molecular weights of the chains at the surface decrease to a value that they become water soluble, they leave the sample surface and mass loss starts. This marks the start of the surface erosion.

If the water can diffuse through the entire specimen and reach a homogenous concentration before the polymer chains at the surface erode (*e.g.,* mass loss starts), the specimen undergoes bulk erosion. On the other hand, if the erosion of the surface chains occurs much earlier than the water can reach the entire specimen, the specimen undergoes surface erosion [18]. In this case, the water penetrates only part of the samples and this part is called the reaction zone. The thickness of the reaction zone is the distance that water diffuses into the specimen within a time when the polymer chains at the surface degrade from their original molecular weights into water soluble segments.

Let the number of monomers per chain in the original polymer be *N_0_* (number of bonds = *N_0_* *-1*) and the number of monomers remaining when the segment becomes soluble be *N_E_*. The number of bonds that need to be hydrolyzed in order for the specimen to reach *N_E_* is (*N_0_/N_E_* *- 1*) per chain. It is convenient to express this number in the form of a concentration *C_m_* (*mol/L*):

(5) $$C_{m}=(\frac{1}{N_{E}}-\frac{1}{N_{0}})\rho$$

where (*mol/L*) is the concentration of degradable bonds in a polymer with an infinitely high molecular weight (*N* → ∞). In most cases, one bond reacts with one molecule of water. Therefore, the total amount of water consumed when the erosion starts is also *C_m_*. It should be noted that *C_m_* measures the cumulative consumption of water in the hydrolysis region when mass loss starts. For polylactide, *N_E_* is about 15 [19]. The degradation rate (*R_d_*) is:

(6) $$R_{d}=k_{2}C_{B}C_{s}$$

The bond concentration *C_B_* can be treated as a constant as *N_E_* *> 1.* Then, we can use *k = k_2_C_B_*. The time for the surface chains to degrade into water soluble size (*T_e_*) is [19]:

(7) $$T_{e}=(\frac{1}{N_{E}}-\frac{1}{N_{0}})\rho \cdot \frac{1}{kC_{w}}$$

Within this time, the water can diffuse:

(8) $$W=q\cdot {\left(\frac{D}{k}\right)}^{1/2}$$

Here *q* is a factor accounting for the effects due to all the other parameters. *(D/k)^1/2^* has units of length and is an intrinsic property of the polymer. When the specimen thickness (*W’*) is << *W*, the specimen undergoes bulk erosion. On the other hand, when *W”* >> *W*, the specimen undergoes surface erosion. Hence, the bulk or surface erosion not only depends on the competition between water diffusion and hydrolysis, but also on specimen thickness. For a specific polymer, thicker specimens tend to undergo surface erosion while thinner specimens tend to undergo bulk erosion.

More rigorous mathematical analysis can be done based on a diffusion-reaction equation with a moving boundary condition [19]. The solutions from this analysis include the following:

The reaction zone width L is:

(9) $$L={\left(\frac{1}{1+\tilde{C}}\right)}^{1/2}{\left(D/k\right)}^{1/2}$$

The marching velocity of the reaction zone is:

(10) $$V={\left((1+\tilde{C}\right)}^{1/2}-{\left(\frac{1}{1+\tilde{C}}\right)}^{1/2}){\left(Dk\right)}^{1/2}$$

The induction time for surface to move is:

(11) $$t_{i}=\frac{1}{k\cdot \tilde{C}}=\frac{L}{V}$$

where *C̃* (= *C_S_/C_m_*), *C_m_* is ρ̃*N* *_E_*., ρ is the molar density of polymer bonds in a polymer (mol/L). The critical sample size for the surface to bulk erosion transition can be estimated based on the water diffusion rate and reaction kinetics as listed in Table 2. As shown, in order for a sample of PLA to be surface eroding, the sample thickness has to be larger than 40 mm. This is larger than most of the common uses for PLA. The surface erosion velocity is about 7 μm/day. Therefore, PLA devices are rarely observed to be surface eroding. Polyanhydrides have a smaller critical size, 20 μm. Devices made from this type polymer are likely to be surface eroding. But, the erosion velocity is very high, 4 mm/day; entire samples can disappear within a day. In reality, the mass loss of this type of polymers is mainly controlled by degradation product dissolution. The degradation products of these polymers have low water solubility; they may stay in semi-solid or gel states for extended period of time after degradation finishes. The erosion of this type of polymers is controlled by degradation product release rates. If they are used for drug delivery, the drug release rates are likely controlled by the degradation products instead of the polymer degradation processes.

## 3. Oxidation Degradation

Oxidation is another mechanism that causes polymers to degrade. For polymers implanted in human body, this is usually due to the oxidation by peroxides produced by the body. Production of the oxidative agents is part of the defense action by the human immune system that always tries to remove foreign materials through inflammation reactions and other processes. This section will discuss three points, production of oxidative agents by biological systems, chemistry of oxidation reactions of polymers, and kinetics of oxidation degradation.

### 3.1. Reactive Oxidative Agents in Tissues

**Figure 14.**
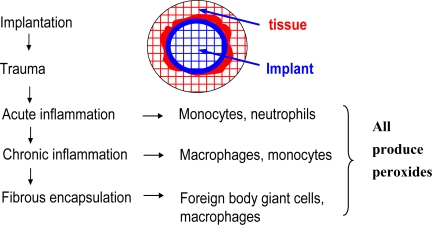
Schematics of inflammatory reactions of tissue to the implants. All the cells, lymphocytes, monocytes, macrophages, and foreign body giant cells produce oxides and release them into the implant.

**Figure 15.**
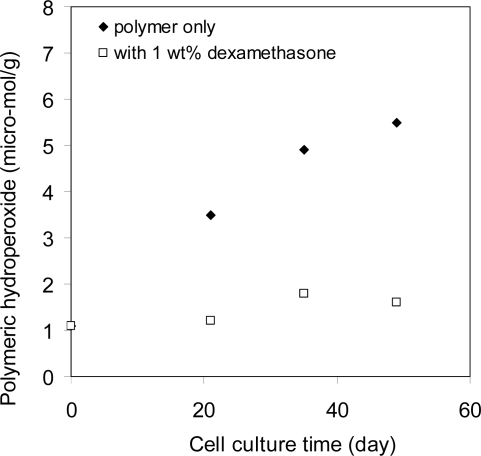
Polymeric hydroperoxide formation in a polyether polyurethane (Pellethane^®^ 80A, Dow). Human monocytes and macrophages were cultured on the surface of the polymer films. The hydroperoxide concentration was measured via an iodometer. The samples were made with either polymer only or the polymer with 1 wt% of dexamethasone (re-plotted based on reference 20).

Upon implantation, inflammatory cells migrate to the implant site, starting a series of events ranging from acute to chronic reactions. For biomaterials that are “accepted” by the body, a capsule forms around the implant which is primarily composed of collagen with foreign body giant cells (FBGC), fibroblasts, and perhaps macrophages inside. The FBGCs are formed by the fusion of macrophages that are differentiated from monocytes (from the acute inflammation reactions). The FBGCs and macrophages produce peroxides that are eluted near the implants with an intent to degrade them (Figure 14). The peroxides produced by the inflammatory cells have been measured experimentally [20]. Figure 15 show the hydroperoxide concentration as a function of time in a polyurethane film on which macrophages were cultured.

In the same experiment, the peroxide concentration was also measured in another polyurethane film that contains an anti-inflammation drug. The peroxide concentration was essentially at the baseline in this film, indicating that the production of peroxides was due to the inflammation reactions. In this experiment, the antioxidant added into the polymer by the manufacturer to control the degradation during processing was removed before the experiment was begun.

### 3.2. Oxidation Chemistry

Certain chemical groups are more susceptible to oxidation reactions than others. Polymers having structures in which free radicals can be easily generated and have reasonable lifetimes are more likely degraded through oxidation reactions. These polymers include polyethylene, polyvinyl, and polyether polyurethanes. All of them have applications in implantable medical products. Polymer having structures in which free radicals cannot be generated are less likely to have oxidation issues. Those polymers include fluoropolymer, silicone, poly(methyl methacrylate), esters, etc. Table 3 lists several commonly used polymers and their oxidation reaction activities.

In oxidation reactions, free radicals are critical, but they act as initiators or reactive intermediate species. The net reaction is between the polymers and oxygen; the products can be water, CO_2_, hydroxyl chain ends, or carboxylic acid chain ends. A general chemical reaction mechanism includes initiation, free radical proliferation, chain transfer, chain breakup, and termination. This is illustrated below.

The initiation processes involves the initial generation of free radicals (Equations 12 and 13). Free radicals can be generated via thermal processes (*e.g.,* during melt-processing), photochemical reactions (during storage and use in light), or radiation reactions (sterilization with gamma radiation or electric beam). In some polymers, these free radicals can quickly decay and disappear but in others they can have relatively long lifetimes and stay in the materials for extended periods. Another source of free radicals is injection by inflammatory cells from the surrounding tissues during foreign body reactions after being implanted.

#### Initiation and Injection

(12) $$I\overset{k_{i}}{\to }I^{•}$$

(13) $$I^{•}+R\overset{k_{iR}}{\to }I+R^{•}$$

Proliferation is the process in which free radicals quickly increase in number by a series reactions with oxygen absorbed in the polymer from the surrounding tissues or blood. The oxygen molecules begin reacting with existing free radicals to produce more radicals (Equation 14 to 15) instead of directly reacting with the polymer groups. The net proliferation reaction is that each oxygen molecule produces two new free radicals (Equation 17). The rate of free radical proliferation is proportional to its own concentration; so it is an accelerated processes. The reaction also depends on oxygen concentration. The limiting step seems to be the peroxide decomposition (Equation 16).

#### Proliferation

(14) $$R_{i}^{•}+O_{2}\overset{k_{ROt}}{\to }R_{i}OO^{•}$$

(15) $$R_{i}OO^{•}+R_{j}H\overset{k_{ORt}}{\to }R_{i}OOH+R_{j}^{•}$$

(16) $$R_{i}OOH\overset{k_{OO_{d}}}{\to }R_{i}O^{•}+HO^{•}$$

#### Net Proliferation

(17) $$RH+O_{2}\overset{k_{RP}}{\to }RO^{•}+HO^{•}$$

In addition to reacting with oxygen molecules to produce more free radicals, the radicals can react with polymer groups to transfer the radicals to different parts of the polymer chains (Equations 18–22). These transfer reactions do not lead to an increase in the number of radicals. But, as will be discussed later, these reactions increase the apparent mobility of free radicals. Also, the transfer reactions are responsible for the formation of small molecular products such as water. Free radicals can also react with themselves to terminate. Such reactions lead to decrease in free radicals (Equations 23 and 24).

#### Transfer

(18) $$R_{i}^{•}+R_{j}H\overset{k_{RRt}}{↔}R_{i}H+R_{j}^{•}$$

(19) $$R_{i}^{•}+R_{j}OH\overset{k_{ROt}}{↔}R_{i}H+R_{j}O^{•}$$

(20) $$R_{i}O^{•}+R_{l}H\overset{k_{OR_{t}}}{\to }R_{i}OH+R_{l}^{•}$$

(21) $$R_{i}O^{•}+R_{j}OH\overset{k_{OOt}}{↔}R_{i}OH+R_{j}O^{•}$$

(22) $$HO^{•}+R_{k}H\overset{k_{OH}}{\to }H_{2}O+R_{k}^{•}$$

#### Termination

(22) $$R_{i}^{•}+R_{j}^{•}\overset{k_{ter}}{\to }R_{i}-R_{j}$$

(23) $$R_{i}^{•}+R_{j}O^{•}\overset{k_{ter}}{\to }R_{i}OR_{j}+h\upsilon$$

However, the most important reactions are those in which the polymer chains are cleaved into shorter segments. These are the degradation steps. Chain break-up occurs at free radical sites (Equations 25 and 26), leading to the formation of two new chain ends, one has a double bond and the other has a free radical. The double bond ends can further react and form acids or ketones. The free radical ends continue through all the possible reactions. The net reaction is one longer chain becomes two short ones. The number of free radicals remains the same (Equation 27).

#### Chain Break up

(25) $$R_{i}^{•}\overset{k_{RB}}{\to }R_{m}C=CH_{2}+R_{n}^{•}$$

(26) $$R_{i}O^{•}\overset{k_{OB}}{\to }R_{m}C=O+R_{n}^{•}$$

*Net chain-break reaction (in combination with a transfer reaction)*

(27) $$R\overset{k_{b}}{\to }R^{\prime }C=X+R^{\prime \prime }$$

where X can be *=CH_2_* or *=O.*

### 3.3. Oxidation Kinetics

In terms of chemistry, the overall oxidation reactions is a break-down of longer polymer chains into shorter ones and conversion of absorbed oxygen into water and other oxidation products. The free radicals act as intermediate species to facilitate these conversions. The total concentration of free radicals increases exponentially during the degradation. Because there are many free radicals and peroxides involved in the reaction it is complicated to develop kinetic equations.

In terms of oxidation kinetics, there are two main mechanisms [21]. The first one is a homogenous reaction mechanism in which all the reactants and products are assumed to have uniform concentrations. The reaction kinetics are controlled by the chemical reactions and diffusion does not affect the distribution of the species. Because the free radicals proliferate as the reactions proceed, the oxidation is autoaccelerated. On the other hand, the rate of free radical termination reactions also increase with increasing radical concentrations; the total radical production rate (initiation, injection, and self-proliferation) will eventually become equal to the total termination rate. Hence, the system reaches a steady state. Quantitative kinetic relationships can be developed based on these assumptions. The results are similar to those for more familiar in low molecular weight hydrocarbons. For example, the rate of the change of peroxides (key component of oxidation reactions) can be described by the following equation:

(28) $$\frac{d[COOH]}{dt}=a\cdot [POOH]-b{\left[POOH\right]}^{2}$$

where, *a* and *b* are rate constants.

This familiar homogenous mechanism has been criticized because it neglects the effects of transport on the oxidation processes [21]. For example, the diffusion coefficient of polymer chains is on the order of 10^−18^ cm^2^/s in the solid state. This is also about the diffusion coefficients of the polymer free radicals that the polymer chains carry. Considering a sample with an initial free radical concentration, [R•], of 10^−6^ M [22], the distance between two radicals is estimated as 100 nm ([1/(10^−6^ × 6 × 10^23^)]^1/3^~100 nm). In order for two radicals to terminate they have to move through diffusion from their average distance (100 nm) to a reaction distance (~0.1 nm). This diffusion would take about 10^8^ s [(100 nm)^2^/10^−18^ cm^2^/s]. Considering a free radical termination reaction rate at k_2_~106 (Ms)^−1^ [23], the termination time can be estimated on the order of 10^0^ s ([R•] × k_2_~10-6 M × 10^6^ (M s)^−1^). Hence, it is extremely unlikely for a reaction to be completed within 1 second while it takes the reactants 10^8^ s to move close enough to react. Also the homogenous mechanism cannot explain why microporous morphology is formed in oxidized polymer samples because microporous morphology suggests a heterogeneous process.

A second mechanism is the heterogeneous model. This model assumes there are zones with higher concentrations of free radicals and zones with lower or zero radical concentration. The oxidation reactions proceeds through free radicals spreading from higher concentration zones to lower concentration zones in a way similar to the spread of diseases [24]. Quantitative kinetic relationships have been developed by using population dynamics with a few adjustable parameters. While this model can qualitatively explain some of the features of heterogeneous reactions, its quantitative kinetics are identical to that of the homogenous mechanism (Equation 28). There is no specific result to support why one should prefer one mechanism over the other. This model does not include molecular mechanisms to explain the free radical spread in terms of chemical reactions and transport processes.

However, the heterogeneous features presented in this radical spread model are reasonable. The authors of the present review propose the following molecular mechanism to explain the free radical spread. Initially, a free radical can do three things: react with an oxygen molecule to produce peroxide and two new radicals; transfer its free electron to another group; or react with other free radicals to terminate. As discussed above, the reaction with other free radicals is a slow process due to diffusion (10^8^ s). However, the transfer reaction with other chemical groups is not limited by diffusion process. This is because the other chemical groups to which the free radicals are transferred are the bulk of the material and have high concentration and close proximity. For example, in poly(tetramethylene oxide) -(CH_2_-CH_2_-CH_2_-CH_2_-O-)_n_, -CH_2_- is the group the free radicals transfer to. Its concentration is about 11 M. The distance between a free radical and neighboring –CH2- groups is on the order of the monomer size (~0.5 nm). There is minimal diffusion required for the transfer reaction. Therefore, the transfer reaction can be estimated based on the reaction without any diffusion required. A typical transfer rate constant is about 10^−2^ M^−1^s^−1^. The reaction time is about 1/([R] k_RRt_)~1/(11 M × 10^−2^ M^−1^s^−1^)~10 seconds. This suggests that every 10 seconds, a free radical transfers to one of its neighbors and moves a distance of a monomer size (0.5 nm). The net effect is the free radical moves in the polymer in a way just like diffusion. The equivalent diffusion coefficient is (0.5 nm)^2^/10s~10^−16^ cm^2^/s. It is interesting that this equivalent diffusion coefficient is orders of magnitude faster than the polymer chain diffusion rate (<10^−18^ cm^2^/s). Most importantly, this diffusion coefficient depends on free radical transfer rates, instead of the condensed state of the polymer. Based on this new “diffusion coefficient,” the termination time would be 10^6^ s.

A free radical’s reaction with an oxygen molecule is not limited by the diffusion process either. The concentration of oxygen in the polymer can be on an order of 10^−3^ M [25], which is equivalent to a molecular distance of 10 nm. The diffusion coefficient of oxygen is about 10^−7^ cm^2^/s [12]. Then, it would take an oxygen molecule (10 nm)^2^/10^−7^(cm^2^/s)~10^−5^ s to diffusion this distance. We assume a free radical reacts with an oxygen molecule at a similar rate as it reacts with a double bond molecule (rate constant 10^2^ M^−1^s^−1^) [23]. Based on the chemical reaction kinetics, the reaction takes about 1/([O_2_] k_Rot_)~1/(10^−3^ × 10^2^)~10 s. The diffusion time is much shorter than this reaction time, therefore the diffusion has minimal effect on the overall kinetics. In order to produce new free radicals, the peroxide radicals have to transfer to other chemical groups (Equation 15) that will decompose into new radicals (Equation 16). Neither of these steps is controlled by diffusion. Therefore, radical proliferation is chemical reaction controlled. However, the slowest reaction probably is the decomposition reaction that can take a time t_0_~104 s [23].

Consider a sample with an initial free radical concentration of 10^−6^ M. Before a radical can terminate with another radical, it can react with oxygen molecules about 10^6^ s/10^4^ s~100 times; the total number of radicals triples each time. However, due to the slow diffusion rates, most of these radical (3^100^) stay around the initial one and form a clone with an increasing volume determined by the diffusion process (~t^3/2^). Therefore, the concentration of free radicals inside the clone increases exponentially with time (~*e^t^*^/^*^t^*^0^ t^−3/2^). Each initial free radical can form a clone and the whole sample contains many individual clones dispersed in the samples. The free radicals react with each other and terminate. This termination allow homogeneous kinetics. This picture explains the origin of the infected zones in the epidemic model. The fraction of oxidized monomers can be estimated based on the reaction and diffusion processes above.

### 3.4. Oxidation Degradation in Polymer Blends and Composites

**Figure 16.**
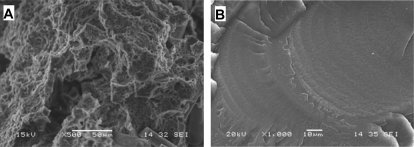
Morphologies of the cross-section (cryo-fracture) of a polyether polyurethane/glass fiber composites after soaked in the H_2_O_2_/CoCl_2_ solution for 56 weeks (A). Microporosity forms in the polymer matrix due to oxidation. For comparison, the cross-section morphology of pure polyurethane samples that were soaked in H_2_O_2_/CoCl_2_ solution for 56 weeks is showed in B.

Oxidation is controlled by chemical reactions, diffusion, or both. Both of these processes are determined by the physical properties of the materials. In multiphase polymer blends or composites, the individual components form separate domains and their physical and chemical properties are the same as they would be alone. Therefore, the oxidation degradation should be the same as in the homopolymers. However, it has been reported in reference [26] that the oxidative degradation of polyether polyurethane was more extensive in composites with glass and carbon fibers than in the pure polymers. As indicated in Figure 16 the polymer matrix of the composites became porous after soaking in an oxidative testing medium (20% H_2_O_2_ aqueous solutions with 0.1 M CoCl_2_). The pure polymer was soaked in the same solution for a same period, but its morphology was only slightly changed. Why was the same polymer oxidized much more severely in the composites than in the pure form? This may be related to the interface of the composites. It has been observed that H_2_O_2_ decomposes much faster when there is glass wool, glass fiber, carbon fiber, or other small particulates present [26,27]. Because this decomposition occurs regardless of the filler material, it seems the surface of the fillers accelerates the peroxide decomposition in the vicinity of interfaces. As suggested by Equation 14, oxygen concentration can speed up the formation of polymer peroxides and production of new free radicals in local areas around the interfaces. As a result, the degradation rates can be greatly accelerated and also a porous structure is formed.

### 3.5. Examples of Oxidation Induced Degradation and Test Methods

Oxidation is detrimental to polymer reliability. One example is the oxidation induced stress cracking of polyether polyurethane in pacemaker lead insulation. The polyether polyurethane is a copolymer made through condensation of polytetramethyleneoxide (PTMO), methylene diphenyl- diisocyanate and (MDI), and 1,4-butanediol (BDO). The polymer grade originally used for lead insulation was made using a high content of PTMO oligomer (1000 kg/mol). As mentioned in the beginning of this section, the hydrocarbon groups next to ether groups are susceptible to peroxide reaction. The PTMO in the insulation was oxidized by the peroxidant produced by the human body. This degradation together with the mechanical stress in the materials resulted in cracking of the polymer surface, which eventually led to a insulation failure.

This problem was solved via engineering the polymer. Because the major component of this polyurethane susceptible to oxidation is the PTMO, polyurethane with less PTMO or another different component that is more stable to oxidation, such as silicone were designed. Such new polyurethanes have been tested and the results are promising [28].

Reliable tests for biological oxidation degradation include *in vivo* evaluations; implanting the devices or materials in the relevant tissue and characterizing the explants in terms of surface and bulk degradation at molecular, microscopic, and macroscopic levels. Identifying and understanding the mechanisms is more important than determining the degree of macroscopic damages. However, *in vivo* tests take time and are expensive. *In vitro* test methods have been developed by a few groups. One of the methods is to soak the test samples in a concentration H_2_O_2_/CoCl_2_ aqueous solution. It was argued that this method mimics the *in vivo* oxidation reaction [27].

## 4. Physical Deterioration Due to Water Absorption

### 4.1. Water Induced-Swelling and Plasticization of Polymers

Most polymers, except polyethylene, polypropylene, and other polyolefins, absorb water. If polymers are used in a dry environment this may not be an issue. However, implantable polymeric materials usually contact tissues whose major component is water; water absorption is a critical factor that determines their performance under use conditions. Absorbed water can affect the performance of polymers in several ways. First, absorbed water is a plasticizer of the polymers. The most significant effect is that the glass transition temperatures of polymers are reduced. The Tg reduction is especially profound in polymers where hydrogen bonding plays important roles such as polyamides, polyurethanes, epoxies, etc. As a result of the Tg reduction, their rigidity is reduced and so is creep resistance. For example, polyether polyurethane (example, Dow 2363-75D) can absorb about 1 wt% water. The tensile modulus of the water soaked polymer can be reduced by almost 50% compared to well dried samples. Polyamide 66’s modulus can be reduced by almost 50% as well after saturation by humid air (relative humidity > 50%). Polyolefin, silicone rubber, and polylactide do not have significant changes in modulus due to the absorption of water. But their long term mechanical stability may still be affected. For example, creep resistance of polylactide is reduced in water.

Absorbed water can accelerate the environmental stress cracking of amorphous polymers such as polycarbonate and polysulfone. These high performance polymers often do not have very high molecular weights. They do not have crystalline domains either. Therefore, their chain molecules cannot be anchored very tightly. Absorbed water molecules reduce the already weakened molecular anchoring. Under chronic stress, chain molecules are pulled out resulting in the formation of micro-cracks. Coalescence of the micro-cracks leads to crack growth and eventual material failure.

### 4.2. Delamination

**Figure 17.**
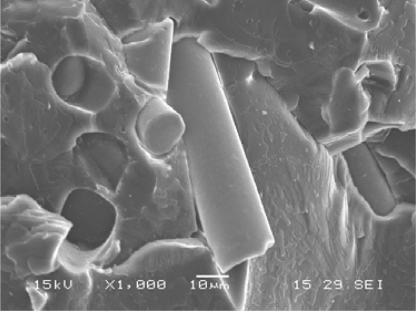
Interfacial delamination in polyurethane/glass fiber composite soaked in phosphate buffered solution (pH 7). The glass fiber delaminated from the polyurethane matrix and the improvement of the mechanical properties of the composite was lost.

Absorbed water has a unique and significant effect on multiphase polymer composites and blends. Water molecules can accumulate at the interface between two phases and cause delamination of the two domains. Composite materials often have combined or synergistic properties, which requires different components of composites to bond strongly. When the two components are hydrophilic, the absorbed water tends to enrich at the interface, and the components may delaminate. Consequently, the composites may lose all the combined or synergistic properties. Figure 17 shows a composite composed of polyurethane and untreated glass fiber. The modulus of the composite is increased by almost 30% when the composite is dry. But when the composite was immersed in water, this improvement was completely lost.

## 5. Applications of Polymers Based on Degradation Properties

### 5.1. Stable Polymers

Chronic implantable medical devices need stable polymers. The polymers used in these devices are required to have acceptable performance for many years or even the lifetime of patients. For example, leads are used to deliver the electric stimulation from electric stimulators (*e.g.,* pacemakers) to the tissues that they treat. The leads are composed of conducting wires and polymeric insulation layers. The lifetime of the insulation layers is ideally the lifetime of the patients. Polyether polyurethane and silicone are the two most commonly used polymers for the insulation. Polyethylene was tried initially, but it was abandoned due to its susceptibility to oxidation degradation. Table 4 lists several commonly used stable polymers and the relevant medical devices.

### 5.2. Degradable Polymers

Not all implantable medical devices require stable polymers. Many devices need biodegradable polymers. For example, polymer depot based drug delivery devices require the polymer matrices to degrade and disappear after the drugs are released. Polylactide, polyanhydrides, collagen, etc, have been widely used for this purpose. Degradable suture is another example. Other examples include surgical adhesives, surgical void fillers, and some orthopedic implants such as rods, etc. Some commonly used degradable polymers and relevant devices are also listed in Table 4.

## 6. Effects of Degradation Products on the Body

An important question to consider is the effect of the polymer degradation products on the body. Hydrolysis produces carboxylic acid and/or hydroxyl chain ends. Hydroxyl groups may be further oxidized later. Oxidation reactions may produce many different species, but they are usually aldehydes, ketones or carboxylic acids. Whether these entities are detrimental to device performance depends on the specific species, amounts, generation rates, and contacting tissues. An implant of small size and slow degradation rate may trigger very mild biological reactions compared to a large implant made of the same material. This issue of concern is biocompatibility. A polymer that does not react with its surroundings (that is, one that would be termed as “inert”) is usually reasonably biostable and may have less biocompatibility concerns. However, it must be remembered that biocompatibility can only be determined under the conditions of use. That is, in the form of the device that will be implanted, and in the tissue it will be used in. Hence, one must be careful in making generalizations about material compatibility.

When a material degrades, it is no longer the material that was originally proven biocompatible. Because of this, the makers of implantable materials must consider stability as one of their biocompatibility criteria. One of the likely scenarios is that the formation of small chain carboxylic acids leads to local pH changes that cause an inflammatory response. The ability of the body to tolerate such a situation depends on a number of things including whether the body can dilute the response through either homeostasis or by simply moving the offending species to somewhere they can be further degraded or flushed out via the kidneys. Rather obviously, if the degrading material is captured in a confined space (such as in a partially healed implant) it may more easily cause an inflammatory reaction.

One often hears concerns about the biocompatibility or toxicity of the parent monomers for the polymer in question. This is particularly true for methylene diamine used to make some polyurethanes. The biggest concern for such cases is not because of degradation since few polymer formation reactions are reversible, and the hydrolysis and oxidation mechanisms discussed here usually do not produce the starting materials. Rather, this concern has more to do with the conditions of synthesis, particularly the synthesis stoichiometry, efficiency of mixing, polymerization extent and post purification. Either of these can produce a situation where there are residual monomers. Such materials may be less biocompatible (more toxic) than optimally synthesized and purified materials. In any case, such issues are not the subject of this manuscript.

## 7. Conclusions

Polymers primarily degrade via hydrolysis and oxidation mechanisms. In most cases these degrading processes are undesirable as they compromise the performance of implantable devices. However, in some cases degradation is an intended function of the device; drug delivery devices that erode and disappear after delivering their drug to the body are examples.

Hydrolysis is fundamentally a second order reaction that depends upon the concentration of attackable bonds and water. Since water is soluble in many polymers on the order of 1% by weight, this helps determine the observed rate of degradation. In some cases the rate of water intrusion into the polymer is much slower than the reaction rate to break the polymer chains into soluble fragments. In such situations the polymer will degrade by surface erosion. Whether surface erosion is observed in any given situation also depends on the surface to volume ratio of the sample.

Oxidation is the other fundamental process that degrades polymers. Oxygen is the reactive element that destroys polymer chains, but it can react in the form of many different oxidative species such as peroxides or free radicals. Oxidation mechanisms often involve free radicals that form, proliferate and move within the polymer before ultimately terminating. These reactions can also involve catalysts including natural enzymes produced by the body.

The details of these degradation processes depend on the specific polymers, their molecular weights and other properties. However, the end result is the original long polymer chains become shorter and shorter until they reach a length where they are soluble in the surrounding medium and are removed via solubility. Because the solubility limit is usually only reached after the original chains have been cleaved many times, the mechanical properties of the polymer deteriorate well before the bulk polymer begins to disappear.

The abundance of water and the relentless onslaught of the body to attack foreign materials means that the performance of implanted polymers in the body depends upon not only the choice of the polymer, but also subtle things like processing, additives and mechanical stress under the usage conditions. Hence, it is very important that hydrolysis and oxidation reactions must be well understood when considering use of such materials for implantable use.

## Figures and Tables

**Table 1.** Charge of atoms in different chemical groups calculated with Material Studio^®^.

**Polymer**	**Group**	**Charge/electron**
Very low hydrolytic activity (with low charge and protecting groups)		
Polyolefin	-C^*^H_2_-CH_2_-	−0.11
Polyether	-C^*^H_2_-O-CH_2_-	0.054
Polysulfone (aromatic)	-C^*^-SO_2_-C-	0.073
PDMS	-Si^*^-O-	0.62
Low hydrolytic activity (due to conjugate structures)		
Polycarbonate	-O-C^*^O-O-	0.72
Polyimide	-(CH_2_-C*O)_2_-N-	0.45
Polyurethane	-O-C^*^O-NH-C-	0.63
Polyester (aromatic)	-C^*^O-O-C-	0.60
Polyamide	-C^*^O-NH-C-	0.45
High hydrolytic activity (due to high charge and steric effects)		
Polyanhydride	-(CH_2_-C*O)_2_-O-	0.56
Polyorthoester	-R-C^*^-(O-R)_3_	0.49
Polyketal	-R_2_-C*-(O-R)_2_	0.32
Polyester (aliphatic)	-C^*^O-O-C-	0.56

**Table 2.** The reduced erosion front width *(D/k)^1/2^* of various polymers.

**Polymers**	**k_r_ (1/s)**	**D (cm^2^/s)**	**(D/k)^1/2^**	**(D k)^1/2^**
poly(anhydride)	2E-03	1E-08	20 μm	4 mm/day
poly(ortho ester)	5E-05	1E-08	140 μm	1 mm/day
poly(α-hydroxy ester)	7E-09	1E-07	40 mm	7 μm/day
poly(amide)	3E-13	1E-08	2 m	50 nm/day

**Table 3.** Oxidation reaction possibilities of a few commonly used polymer structures.

**Chemical groups susceptible to oxidation**		**Chemical groups less susceptible to oxidation**	
Polyolefin (*e.g.,* PE)	-CH_2_-^*^CH_2_-CH_2_-	Fluoropolymers	-CF_2_-CF_2_-CF_2_-
Vinyl polymers	-CH_2_-^*^CHR-CH_2_-	Polyesters	-CH(CH_3_)-C(=O)O-
Polyethers	-CH_2_-O-^*^CH_2_-	Methacrylates	-C(CH_3_)(COOCH_3_)CH_2_-
Polyamines	-CH_2_-^*^N-CH_2_-	Silicone	-Si(CH_3_)_2_-O-Si(CH_3_)_2_-O-
		Polysulfone	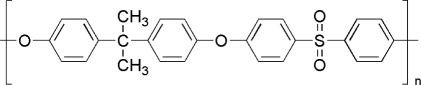
		Polyetheretherketone	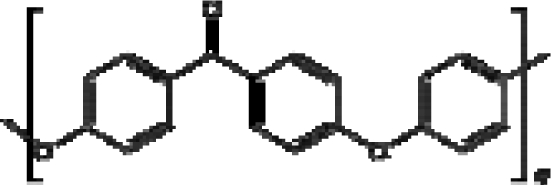

**Table 4.** Polymers used in approved implantable medical devices.

**Polymers**	**Application examples**	**Preferred properties**
Silicone	Catheters, lead insulations, tissue filling, adhesives, intraocular lens	Stable
Polyurethanes (ether, ester, carbonate, hydrocarbon)	Catheters, lead insulations, structural components (*e.g.,* artificial heart), pacemaker connectors, cervical (spinal) disc replacement, sensors	Stable
Polyetheretherketone (PEEK)	Orthopedic parts,	Stable
Polysulfone	Structural components	Stable
Epoxy	Structural components	Stable
Polyethylene	Joint replacement	Stable
Poly(methyl methacrylate)	Bone cement, intraocular lens,	Stable
Fluoropolymer	Vascular grafts, drug delivery coating	Stable
Poly(ethylene-co-vinyl acetate)	Drug delivery	Stable
Poly(ethylene terephthalate)	Vascular grafts, artificial heart valve suture	Stable
Poly(lactide) and its copolymers	Orthopedic products, drug delivery, suture	Degradable
Polyanhydride	Drug delivery	Degradable
Collagen	Tissue filling, tissue engineering scaffolds, drug delivery	Degradable
